# Length of stay following cesarean sections: A population based study in the Friuli Venezia Giulia region (North-Eastern Italy), 2005-2015

**DOI:** 10.1371/journal.pone.0210753

**Published:** 2019-02-27

**Authors:** Luca Cegolon, Giuseppe Mastrangelo, Oona M. Campbell, Manuela Giangreco, Salvatore Alberico, Lorenzo Montasta, Luca Ronfani, Fabio Barbone

**Affiliations:** 1 Institute for Maternal and Child Health, IRCCS “Burlo Garofolo”,Trieste, Italy; 2 Local Health Unit N.2 “Marca Trevigiana”, Public Health Department, Veneto Region, Treviso, Italy; 3 Padua University, Department of Cardio-Thoracic & Vascular Sciences, Padua, Italy; 4 London School of Hygiene & Tropical Medicine, Faculty of Epidemiology & Population Health, MARCH Centre, London, United Kingdom; Federal University of Sergipe, BRAZIL

## Abstract

**Background:**

Births by cesarean section (CS) usually require longer recovery time, and as a result women remain hospitalized longer following CS than vaginal delivery (VD). A number of strategies have been proposed to reduce avoidable health care costs associated with childbirth. Among these, the containment of length of hospital stay (LoS) has been identified as an important quality indicator of obstetric care and performance efficiency of maternity centres. Since improvement of obstetric care at hospital level needs quantitative evidence, we compared the maternity services of an Italian region on LoS post CS.

**Methods:**

We conducted a population-based study in Friuli Venezia Giulia (FVG), a region of North-Eastern Italy, collecting data from all its 12 maternity centres (coded from A to K) during 2005–2015. We fitted a multivariable logistic regression using LoS as a binary outcome, higher/lower than the international early discharge (ED) cutoffs for CS (4 days), controlling for hospitals as well as several factors related to the clinical conditions of the mothers and the newborn, the obstetric history and socio-demographic background. Results were expressed as adjusted odds ratios (aOR) with 95% confidence interval (95%CI). Population attributable risks (PARs) were also calculated as proportional variation of LoS>ED for each hospital in the ideal scenario of having the same performance as centre J (the reference) during calendar year 2015. Results were expressed as PAR with 95%CI. Differences in mean LoS were also investigated with a multivariable linear regression model including the same explanatory factors of the above multiple logistic regression. Results were expressed as adjusted regression coefficients (aRC) with 95%CI.

**Results:**

Although decreasing over the years (5.0 ± 1.7 days in 2005 vs. 4.4 ± 1.7 days in 2015), the pooled mean LoS in the whole FVG during these 11 years was still 4.7 ± 1.7 days, higher than respective international ED benchmark. The significant decreasing trend of LoS>ED over time in FVG (aOR = 0.89; 95%CI: 0.88; 0.90) was marginal as compared to the variability of LoS>ED observed among the various maternity services. Regardless it was expressed as aRC or aOR, LoS after CS was lowest in hospital C, highest in hospital D and intermediate in centres I, K, G, F, A, H, E, B and J (in descending order). The aOR of LoS being longer than ED ranged from 1.63 (95%CI:1.46; 1.81) in hospital B up to 32.09 (95%CI: 25.68; 40.10) in facility D. When hospitals were ranked by PAR the same pattern was found, even if restricting the analysis to low risk pregnancies.

**Conclusions:**

Although significantly decreasing over time, the mean LoS in FVG during 2005–2015 was 4.7 days, higher than the international threshold recommended for CS. There was substantial variability in LoS by facility centre, suggesting that internal organizational processes of single hospitals should be improved by enforcing standardized guidelines and using audits, economic incentives and penalties if need be.

## Background

Cesarean sections (CS) are among the most common and long-standing obstetric surgical procedures worldwide, employed when vaginal deliveries (VD) are impossible or in case of life threatening risks for the mother and/or the newborn [1–4]. Although, CS are pushed also by a number of other factors including maternal request, fear of medico-legal backlashes, economic convenience and social/cultural trends [5–9]. Nonetheless, CSs entail health risks for the mother and the newborn, such as surgical site infections, venous thrombo-embolism, shock, hemorrhage, early childhood anemia. Moreoveor, births by CS usually require longer time to recovery [9–11], and as a result, women remain hospitalized longer following CS than VD, with subsequent considerable enhancement of health care costs [12–14].

A number of strategies have been proposed to reduce avoidable health care costs associated with childbirth [4,15,16]. Among these, length of hospital stay (LoS) has been identified as an important quality indicator of obstetric care and efficiency of hospital performance [17–21]. All else being equal, shorter LoS would reduce hospital charges for patients and allow them faster return home [22].

Early discharge (ED) following childbirth is a concept that has been increasingly introduced to improve quality of care and in response to budget constraints, higher patients’ needs and safety of care [23]. The definition of ED most frequently employed worldwide is the one proposed by the American Academy of Pediatrics and the American College of Obstetricians and Gynecologists: LoS less than 48h (2 days) for spontaneous VD and less than 96h (4 days) post CS [17,21,24].

The World Health Organization (WHO) has recently recommended evaluation of health care services for the appropriate use of available resources. An evidence-based appraisal should be conducted “*at hospital level*” and “*in a standardized and action-oriented manner*, *with the inclusion of maternal and perinatal outcomes*”, in order to be able to provide adequate conclusions to format policies, practices and actions [25].

Using such approach, in a previous study we investigated hospital performance in terms of LoS following VD (spontaneous as well as instrumental deliveries) in Friuli-Venezia Giulia (FVG), a region of North-Eastern Italy with a population of approximately 1.22 million residents, 50% of whom are females [17].

Since evaluation of obstetric care at hospital level should be evidence-based, we conducted the present study comparing the maternity services of the above Italian region (FVG) on LoS after CS in order to inform policy makers. Containment of unnecessary extended LoS by enforcing standardized practice patterns could contribute to improve the efficiency of maternity services.

## Methods

The methods have been reported in a previous paper [17] and are herewith briefly described.

### Study design

This is a population-based cross-sectional study to investigate LoS after CS during 2005–2015 in FVG. The study protocol was approved by the Regional Health Authority of FVG. Data analyzed in this study were fully anonymized before being accessed, hence informed consent from patients was waived from the Regional Health Authority of Friuli Venezia Giulia.

### The database

**Fig 1:**
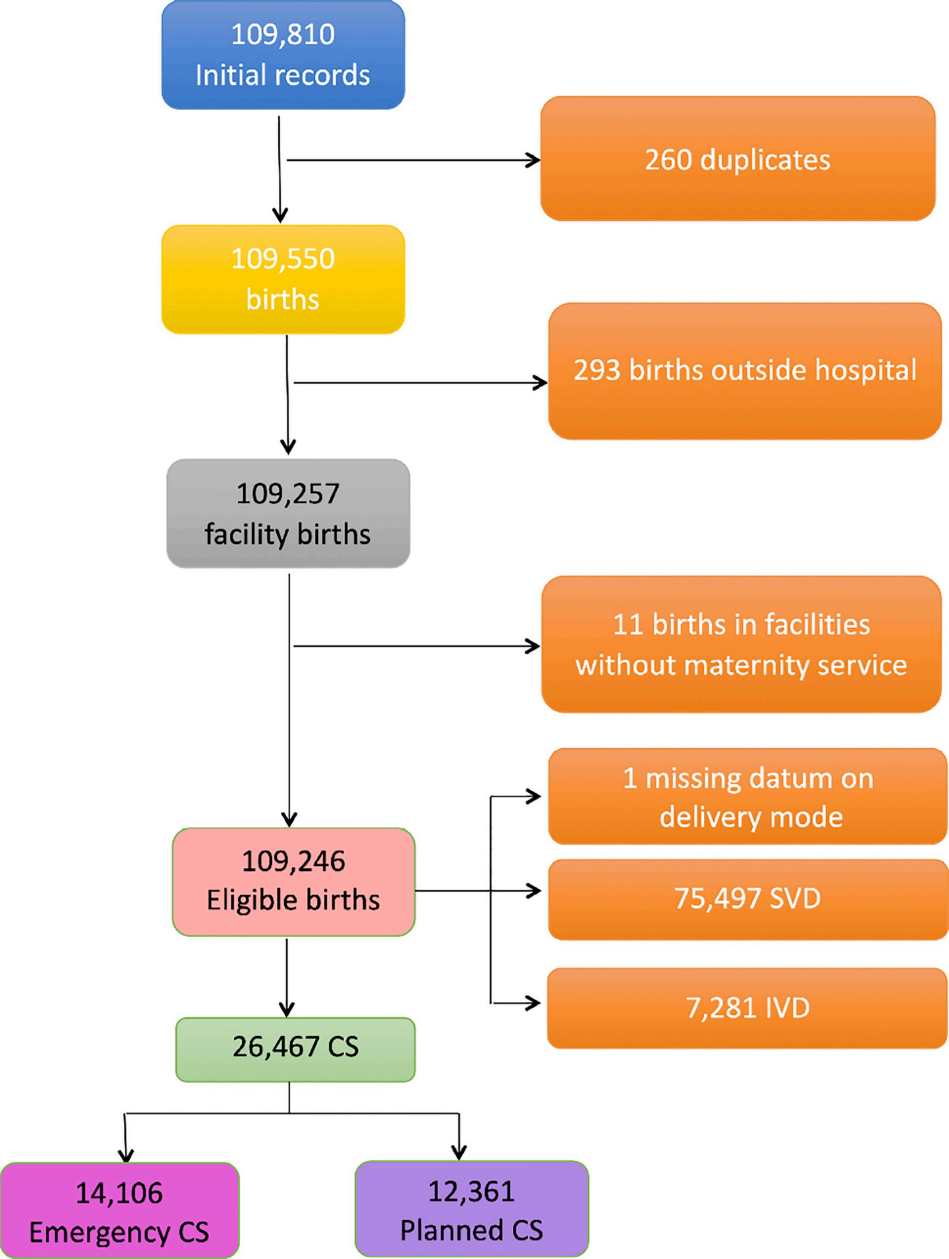
Flowchart displaying the various criteria applied to the initial database to obtain the final number of hospital records available for the analysis.

We used hospital discharge forms from 2005 to 2015 as well as data collected by the Certificate of Delivery Care (CEDAP, S1 Fig). CEDAP is a formatted questionnaire collecting clinical and personal information on mothers and newborn [17]. The 12 regional facility centres were anonymized and named by alphabetic letters from A to L. The criteria applied to the initial database to obtain the final number of CSs are shown in Fig 1.

### Length of stay

LoS (measured in days) was calculated by subtracting the date of birth from the date of hospital discharge. We considered the average LoS and the percentage of LoS > ED benchmark following CS (4 days).

**Fig 2:**
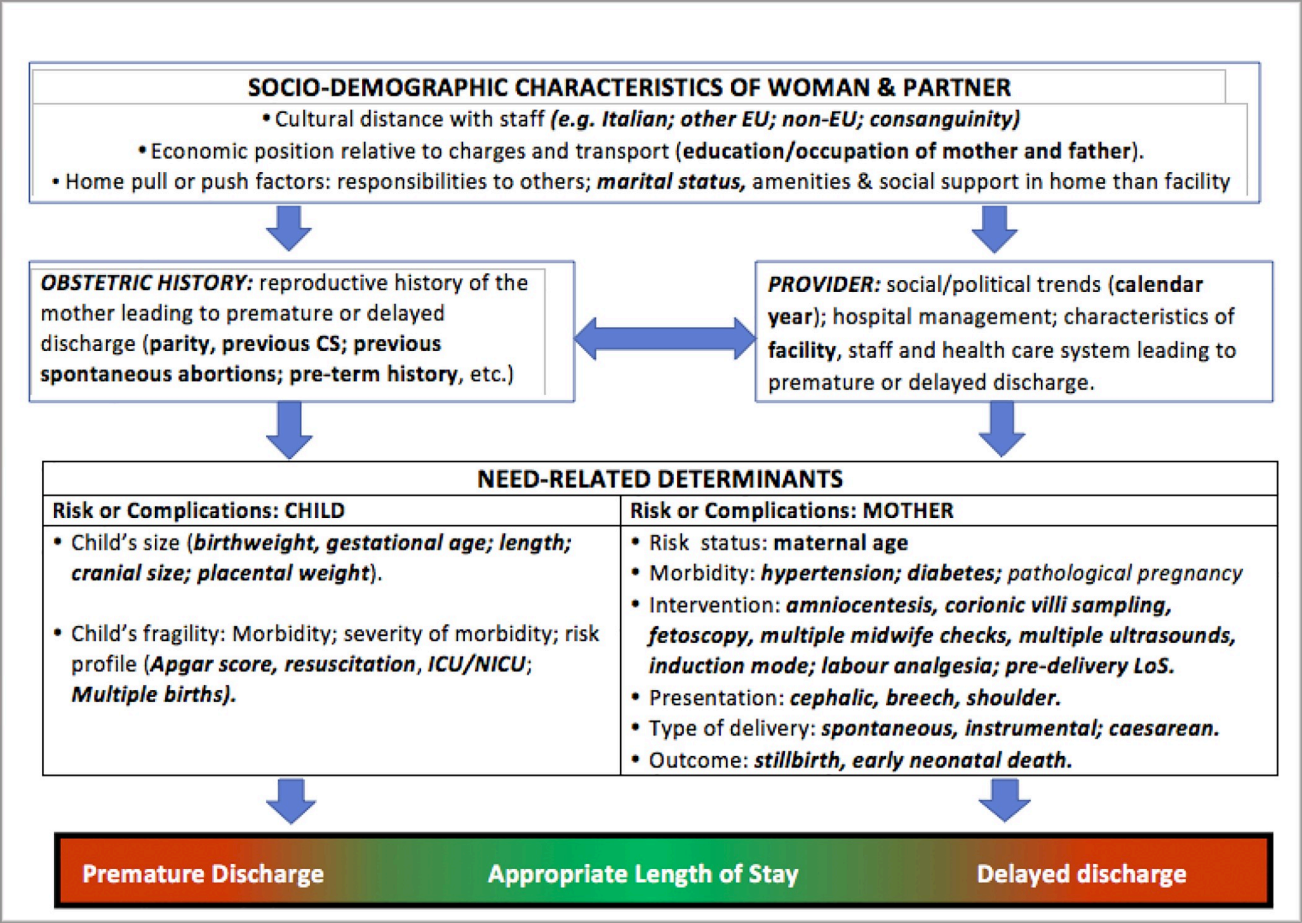
Conceptual framework explaining the relationship between various determinants and LoS.

We employed the conceptual framework already adopted [17], identifying five broad domains of potential determinants of LoS (Fig 2).

1 **Health care setting and time frame:** hospitals and calendar year (Table 1).

**Table 1:** Distribution of length of stay (LoS, in days) after cesarean section (CS) by calendar year and hospital: number (N), percentage (%); mean LoS ± standard deviation (SD; Mis^a^ = missing values on all births; Mis^b^ = missing values considering only CS.

FACTORS	STRATA	ALL BIRTHS (N = 109,246)	CESAREAN SECTIONS (N = 26,467)								
			Number (Row %)	LoS (days)							
				Mean ± SD	≤1	2	3	4	5	6+	>4
					Row %						
**Calendar** **Year**	**2005**	10,173	2,527 (24.8)	5.0 ± 1.7	0.0	0.5	7.3	34.0	34.5	23.7	58.2
	**2006**	10,468	2,615 (25.0)	4.8 ± 1.5	0.1	0.7	9.5	38.6	27.7	23.4	51.1
	**2007**	10,652	2,700 (25.4)	4.7 ± 1.5	0.1	0.7	10.7	42.8	25.9	19.7	45.6
	**2008**	10,478	2,571 (24.5)	4.8 ± 1.6	0.1	0.6	8.8	44.4	25.8	20.3	46.1
	**2009**	10,492	2,679 (25.5)	4.7 ± 1.7	0.0	0.5	10.4	44.8	25.6	18.6	44.3
	**2010**	10,406	2,547 (24.5)	4.7 ± 1.6	0.1	0.4	11.5	45.3	25.5	17.2	42.7
	**2011**	9,791	2,353 (24.0)	4.7 ± 1.8	0.1	0.6	12.2	46.0	24.3	16.8	41.1
	**2012**	9,743	2,154 (22.1)	4.5 ± 1.5	0.1	0.6	13.8	47.8	23.1	14.7	37.8
	**2013**	9,289	2,225 (24.0)	4.6 ± 1.8	0.1	1.0	14.0	48.5	21.5	14.9	36.4
	**2014**	9,095	2,090 (23.0)	4.5 ± 1.9	0.2	0.8	16.0	49.2	20.2	13.6	33.8
	**2015**	8,659	2,006 (23.2)	4.4 ± 1.7	0.1	0.4	20.0	51.7	14.8	13.1	27.9
**TOTAL**		**109,246**	**26,467 (24.2)**	**4.7** ± 1.7	**0.1**	**0.6**	**11.9**	**44.5**	**24.8**	**18.1**	**42.9**
**Hospital** (Mis^a^: 193) (Mis^b^: 71)	**A**	19,059	4,430 (23.2)	5.0 ± 1.8	0.1	0.9	5.2	48.0	8.3	37.5	45.9
	**B**	18,380	6,307 (34.3)	4.6 ± 1.6	0.0	0.2	5.3	59.7	20.9	14.0	34.8
	**C**	8,840	1,797 (20.1)	4.3 ± 1.2	0.0	0.2	4.5	81.8	7.8	5.7	13.5
	**D**	3,330	942 (28.3)	5.5 ± 1.4	0.1	0.2	0.9	11.5	46.4	41.0	87.3
	**E**	6,673	1,628 (24.4)	4.7 ± 1.5	0.1	0.4	4.3	63.3	7.2	24.8	31.9
	**F**	5,723	1,469 (25.7)	4.7 ± 1.1	0.0	0.6	6.9	32.7	52.7	7.2	59.9
	**G**	9,146	1,386 (15.2)	4.8 ± 1.3	0.0	0.8	4.9	33.9	50.0	10.4	60.4
	**H**	11,681	1,920 (16.4)	4.2 ± 1.9	0.1	1.8	31.0	31.1	28.8	7.2	36.0
	**I**	6,047	1,303 (21.6)	5.1 ± 1.3	0.1	0.1	2.5	17.7	61.5	18.0	79.6
	**J**	12,035	3,461 (28.8)	4.3 ± 2.2	0.2	1.3	43.8	29.4	10.5	14.9	25.3
	**K**	8,027	1,741 (21.7)	4.8 ± 1.2	0.1	0.2	6.0	25.5	56.5	11.8	68.3
	**L**	12	12 (100.0)	4.9 ± 4.9	33.3	8.3	8.3	8.3	0	41.7	41.7
**TOTAL**		**109,053**	**26,396 (24.2)**	**4.7** ± 1.7	**0.1**	**0.6**	**11.9**	**44.5**	**24.8**	**18.1**	**42.9**

2 **Maternal health factors** (Table 2): mother’s age, hypertension/diabetes, amniocentesis, villi samples, fetoscopy, pre-delivery LoS, presentation, labour induction, labour analgesia, neonatal status, number of obstetric checks performed in pregnancy, number of ultrasound (US) scans performed during pregnancy.

**Table 2:** Distribution of length of stay (LoS, in days) after cesarean section (CS) by maternal health factors. Number (N), percentage (%); mean LoS ± standard deviation (SD; Mis a = missing values on all births; Mis b: missing values considering only CS.

FACTORS		STRATA	ALL BIRTHS (N = 109,246)	CESAREAN SECTIONS (N = 26,467)								
				Number (Row %)	LoS (days)							
					Mean ± SD	≤1	2	3	4	5	6+	>4
						Row %						
**Cesarean type**		Urgent		14,106 (53.3)^**@**^	4.8 ±1.8	0.1	0.6	11.0	42.7	25.7	19.6	45.7
		Planned		12,361 (46.7)^**@**^	4.5 ± 1.5	0.1	0.7	12.9	46.5	23.8	16.0	39.8
**Mother Age** (years) (Mis a: 32; Mis b: 12)		15–19	1,254	193 (15.4)	4.9 ± 1.7	0.0	1.1	8.4	42.4	24.6	23.6	48.2
		20–24	9,485	1,688 (17.8)	4.8 ± 2.1	0.0	0.6	11.8	44.6	23.5	19.5	43.0
		25–29	23,675	4,879 (20.6)	4.6 ± 1.7	0.1	0.8	12.7	44.9	24.5	17.1	41.5
		30–34	38,381	9,033 (23.5)	4.6 ± 1.5	0.1	0.8	12.5	44.2	26.0	16.5	42.5
		35–39	28,860	7,943 (27.5)	4.7 ± 1.7	0.1	0.4	11.3	45.3	24.1	18.9	43.0
		40–44	7,214	2,525 (35.0)	4.8 ± 1.7	0.0	0.4	11.1	43.3	24.2	20.9	45.1
		45+	345	194 (56.2)	5.3 ± 2.6	0.0	0.0	5.8	33.3	28.0	32.8	60.9
**Hypertension/** **diabetes** (Mis a: 63; Mis b: 14)		No	106,690	25,173 23.6)	4.6 ± 1.6	0.1	0.6	12.1	45.3	24.9	17.0	41.9
		Yes	2,493	1,280 (51.3)	5.6 ± 2.6	0.2	0.2	8.1	27.7	23.9	39.9	63.8
**Villi sample** **(**Mis a: 6; Mis b:3)		No	104,993	25,125 (24.0)	4.7 ± 1.7	0.1	0.6	11.9	44.6	24.5	18.2	42.8
		Yes	4,247	1,249 (29.4)	4.6 ± 1.5	0.1	0.3	11.9	42.0	30.0	15.7	45.7
**Amniocentesis** (Mis a: 6; Mis b:3)		No	91,986	21,376 (23.2)	4.7 ± 1.6	0.1	0.7	12.2	44.7	25.0	17.5	42.4
		Yes	17,254	5,088 (29.5)	4.7 ± 1.6	0.1	0.4	10.7	43.7	24.2	20.9	45.1
**Fetoscopy** (Mis: 6; Mis b: 3)		No	108,892	26,369 24.2)	4.7 ± 1.7	0.1	0.6	11.9	44.5	24.8	18.1	42.9
		Yes	348	95 (27.3)	4.7 ± 1.4	0.0	1.1	8.6	46.2	23.7	20.4	44.1
**N. obstetric** **checks in pregnancy** (Mis a: 1; Mis b: 0)		<4	20,856	5,592 (26.8)	5.0 ± 1.8	0.1	0.6	8.4	37.8	27.0	26.2	53.1
		4–7	65,800	15,145 (23.0)	4.6 ± 1.7	0.1	0.6	13.3	46.6	25.3	14.1	39.4
		8+	22,589	5,730 25.4)	4.7 ± 1.6	0.1	0.6	11.6	45.6	21.3	20.9	42.1
**N. US scans** **in pregnancy** (Mis a: 7; Mis B: 2)		<4	19,003	3,305 (17.4)	4.7 ± 1.8	0.1	1.0	15.1	42.9	22.8	18.2	41.0
		4–5	52,873	11,686 (22.1)	4.6 ± 1.6	0.1	0.6	13.5	45.7	25.2	14.9	40.1
		6+	37,363	11,474 30.7)	4.8 ± 1.7	0.1	0.5	9.3	43.8	24.9	21.4	46.3
**Labour analgesia** (Mis a: 184; Mis b: 127)		No	89,536	23,111 (25.8)	4.7 ± 1.7	0.1	0.6	12.2	43.6	24.9	18.6	43.5
		Yes	19,526	3,229 16.5)	4.6 ± 1.5	0.1	0.7	9.1	50.7	24.7	14.8	39.5
**Labour induction** (Mis a: 68; Mis b: 15)		No	81,859	25,782 (0.8)	4.4 ± 1.6	0.2	6.8	20.6	32.7	22.7	17.1	39.8
		Yes	27,319	670 (94.4)	4.7 ± 1.7	0.1	0.5	11.7	44.8	24.9	18.2	43.0
**Neonatal status**		Liveborn	108,944	26,377 24.2)	4.7 ± 1.7	0.1	0.6	11.9	44.5	24.9	18.1	42.9
		Stillborn	302	90 (29.8)	5.4 ± 3.5	0.0	5.6	24.4	27.8	11.1	31.1	42.2
**Pre-delivery LoS** (Mis a:594; Mis b: 184)		<3 days	103,769	23,583 (22.7)	4.6 ± 1.5	0.1	0.6	11.9	45.8	25.2	16.3	41.5
		3–5 days	3,142	1,489 (47.4)	5.1 ± 2.2	0.1	0.5	11.8	38.0	21.2	28.3	49.6
		6+ days	1,741	1,211 (69.6)	5.7 ± 2.9	0.1	0.5	11.2	26.7	20.8	40.8	61.6
**Presentation** (Mis a: 181; Mis b: 164)	**Cefalic**	Spontaneous	75,118		2.9 ± 1.1							
		Instrumental	7,248		3.3 ± 1.3							
		Cesarean	21,284	21,284 (20.5)	4.7 ± 1.7	0.1	0.7	12.3	45.3	24.0	17.7	41.7
	**Breech**	Spontaneous	368		3.0 ± 1.4							
		Instrumental	27		3.8 ± 1.6							
		Cesarean	4,893	4,983 (92.5)	4.7 ± 1.6	0.1	0.4	10.2	42.0	28.3	19.1	47.4
	**Shoulder**	Spontaneous	0									
		Instrumental	0									
		Cesarean	126	126 (100.0)	5.3 ± 2.4	0.0	0.8	10.4	29.6	26.4	32.8	59.2

@: column percentages.

3 **Clinical factors of the child** (Table 3), and in particular:
  3.1 **Child’s size factors**: gestational age; birthweight; placenta weight; and a variable “child’s size” created combining the distribution of four factors: sex of child; parity; birth-weight and gestational age. The variable “child’s size” enabled to classify newborn into small for gestational age (SGA); appropriate for gestational age (AGA); large for gestational age (LGA) [17, 26,27].
  3.2 **Child’s fragility factors:** Apgar score at 1 minute; Apgar score at 5 minutes; resuscitation; intensive care unit (ICU) admission; multiple birth.

**Table 3:** Distribution of length of stay (LoS, in days) after cesarean section (CS) by clinical factors of the newborn. Number (N), percentage (%); mean LoS ± standard deviation (SD); SGA = Small for gestationl age; AGA = appropriate for gestational age; LGA = large for gestational age. Mis a = missing values on all births; Mis b = Missing values considering only CS.

FACTORS	STRATA		ALL BIRTHS (N = 109,246)	CESAREAN SECTIONS (N = 26,467)								
				Number (Row %)	LoS (days)							
					Mean ± SD	≤1	2	3	4	5	6+	>4
						Row %						
**CHILD’S SIZE FACTORS**												
**Gestational** **Age** (weeks)	<29		563	369 (65.5)	5.7 ± 3.1	0.0	1.1	13.4	34.5	13.4	37.5	51.0
	29–32		1,130	855 (75.7)	5.2 ± 2.4	0.7	0.7	9.9	38.3	17.3	33.1	50.4
	33–36		6,217	3,219 (51.8)	5.5 ± 2.3	0.2	0.5	8.4	26.2	22.7	42.1	64.8
	37–40		82,637	18,535 (22.4)	4.5 ± 1.4	0.1	0.6	12.3	47.5	25.6	14.0	39.6
	41+		18,699	3,489 (18.7)	4.5 ± 1.5	0.1	0.8	13.5	47.8	25.7	12.2	37.9
**Birthweight** (gr) (Mis a: 5; Mis b = 2)	<1000		525	328 (62.5)	5.4 ± 2.6	0.3	0.6	10.8	34.5	16.7	37.0	53.7
	1,000–1,499		668	548 (82.0)								
	1,500–1,999		1,330	1,018 (76.5)								
	2,000–2,499		4,524	2,275 (50.3)	5.5 ± 2.2	0.2	0.6	6.6	24.6	26.3	41.6	67.9
	2,500–3,999		95,954	20,627 (21.7)	4.5 ± 1.5	0.1	0.6	12.6	47.1	25.4	14.3	39.6
	4,000–4,499		6,576	1,461 (22.2)	4.5 ± 1.5	0	0.4	11.9	50.3	24.7	12.7	37.4
	4,500+		664	208 (31.3)								
**Placenta weight** (gr) (Mis a: 172; Mis b: 83)	<500		22,862	5,473 (23.9)	5.0 ± 2.0	0.2	0.6	9.8	38.7	23.9	26.9	50.7
	500–599		35,744	6,819 (19.1)	4.6 ± 1.4	0.0	0.8	12.3	45.4	25.6	15.9	41.5
	600–999		49,048	12,986 (26.5)	4.5 ± 1.6	0.1	0.6	13.1	47.7	24.6	14.1	38.6
	1,000–1,500		1,420	1,106 (77.9)	5.3 ± 2.1	0.0	0.5	5.7	30.7	27.3	35.8	63.1
Child’s size *	SGA		9,122	2,929 (32.1)	5.0 ± 1.8	0.1	0.7	9.6	36.2	26.5	26.9	53.4
	AGA		88,138	20,479 (23.2)	4.6 ± 1.7	0.1	0.6	12.3	45.2	24.5	17.2	41.8
	LGA		11,986	3,059 (25.5)	4.6 ± 1.6	0.0	0.4	11.3	47.5	25.0	15.7	40.7
**CHILD’S FRAGILITY FACTORS**												
**Apgar** **1 min**	<7		6,807	2,988 (43.9)	5.2 ± 2.5	0.2	0.8	9.9	37.3	22.5	29.3	51.8
	7+		102,439	23,479 (22.9)	4.6 ± 1.5	0.1	0.6	12.2	45.4	25.1	16.7	41.8
**Apgar** **5 min**	<8		2,386	1,160 (48.6)	5.3 ± 2.6	0.6	1.2	11.0	36.4	18.7	32.2	50.8
	8+		106.860	25,307 (23.7)	4.7 ± 1.6	0.1	0.6	11.9	44.9	25.1	17.5	42.6
**ICU adm.** (Mis a: 221; Mis b: 36)	No		103,900	23,250 (22.4)	4.6 ± 1.5	0.1	0.6	12.2	45.8	26.1	15.2	41.3
	Yes		5,125	3,181 (62.1)	5.4 ± 2.5	0.3	0.5	9.4	35.0	15.1	39.6	54.7
**Resuscitation** (Mis a: 54; Mis b: 12)	No		106,774	25,053 (23.5)	4.6 ± 1.6	0.1	0.6	12.0	45.0	25.2	17.2	42.4
	Yes		2,418	1,402 (58.0)	5.4 ± 2.7	0.4	0.6	10.5	35.6	18.0	35.0	53.0
**Multiple births** (Mis a: 898; Mis b: 765)	Singleton	Female	51,806	24,179 (22.7)	4.6 ± 1.6	0.1	0.7	12.6	46.1	24.7	15.9	40.6
		Male	54,797									
	Twins or more		1,745	1,523 (87.3)	5.5 ± 1.9	0.1	0.4	4.9	27.6	26.1	40.9	67.1

4 **Socio-demographic background** (Table 4), namely: mother’s nationality; marital status of the woman; mother’s education; mother’s occupation; father’s age; father’s education; father’s occupation; consanguinity.

**Table 4:** Distribution of length of stay (LoS, in days) after cesarean section (CS) by socio demographic and obstetric history factors. Number (N), percentage (%); mean LoS ± standard deviation (SD); Mis a: missing values on all births; Mis b: missing values considering only CS. Self-e = self-employed.

FACTORS	STRATA		ALL BIRTHS (N = 109,246)	CESAREAN SECTIONS (N = 26,467)								
				Number (Row %)	LoS (days)							
					Mean ± SD	≤1	2	3	4	5	6+	>4
						Row %						
**SOCIO-DEMOGRAPHIC FACTORS**												
**Father’s age** (years) (Mis a: 1,949; Mis b: 495)	15–19		199	20 (10.1)	5.1 ± 1.8	0.0	0.0	10.0	35.0	35.0	20.0	55.0
	20–24		2,798	480 (17.2)	4.7 ± 1.7	0.0	0.2	14.3	42.2	24.8	18.5	43.3
	25–29		12,982	2,696 (20.8)	4.7 ± 1.8	0.1	0.7	12.1	45.5	23.9	17.7	41.6
	30–34		31,601	7,168 (22.7	4.6 ± 1.6	0.1	0.9	13.0	44.6	25.2	16.2	41.4
	35–39		34,560	8,478 (24.5)	4.7 ± 1.6	0.1	0.5	11.9	44.5	25.1	17.9	43.0
	40–44		17,866	4,898 (27.4)	4.7 ± 1.7	0.1	0.6	11.2	44.3	24.6	19.3	43.9
	45–49		5,353	1,632 (30.5)	4.7 ± 1.5	0.0	0.5	11.3	44.6	23.7	20.0	43.7
	50–54		1,361	420 (30.9)	4.9 ± 1.9	0.0	8.0	8.0	43.0	26.5	21.6	48.1
	55+		577	180 (31.2)	4.8 ± 1.6	0.0	1.1	7.8	45.6	25.6	20.0	45.6
**Mother’s** **nationality** (Mis a:116; Mis b: 36)	EU	Italian	86,083	20,662 (24.0)	4.6 ±1.6	0.1	0.6	12.0	44.7	25.2	17.4	42.6
		Non-Italian	5,983	1,242 (20.8)	4.4 ±1.3	0.2	0.9	14.2	49.0	23.4	12.4	35.8
	Non-EU		17,064	4,527 (26.5)	4.9 ± 2.1	0.1	0.5	10.8	42.4	23.2	23.0	46.2
**Marital status** (Mis a: 8,155; Mis b: 2,068)	Not married		12,036	2,872 (23.9)	4.8 ± 1.8	0.1	0.7	8.7	44.0	24.7	21.8	46.5
	Married		70,340	17,136 (24.4)	4.7 ± 1.7	0.1	0.6	12.6	43.5	24.7	18.5	43.2
	Separated		1,136	606 (32.1)	4.7 ± 2.1	0.2	0.3	13.0	43.2	23.1	20.1	43.2
	Widow		82									
	Divorced		669									
	Living together		16,846	3,785 (22.5)	4.6 ± 1.6	0.0	0.8	13.7	45.4	24.2	16.0	40.2
**Mother’s education** (Mis a: 24; Mis b: 9)	University or more		29,150	6,935 (23.8)	4.7 ± 1.6	0.1	0.7	11.5	45.9	23.0	18.9	41.9
	Secondary		52,988	12,617 (23.8)	4.6 ± 1.6	0.1	0.6	12.4	44.3	25.6	17.1	42.7
	Junior Secondary		25,107	6,347 (25.3)	4.7 ± 1.9	0.1	0.6	11.8	43.4	25.3	18.7	44.0
	Primary/none		1,977	559 (28.3)	5.0 ± 2.0	0.0	0.9	7.8	43.2	22.9	25.2	48.1
**Father’s education** (Mis a: 6,772; Mis b: 1,798)	University or more		18,542	4,527 (24.4)	4.6 ± 1.6	0.1	0.8	12.1	46.3	21.5	19.2	40.7
	Secondary		51,356	12,156 (23.7)	4.6 ± 1.6	0.1	0.5	13.1	44.9	23.9	17.5	41.4
	Junior Secondary		30,767	7,510 (24.4)	4.7 ± 1.8	0.1	0.7	11.2	45.3	23.6	19.1	42.8
	Primary/none		1,809	476 (26.3)	4.9 ± 2.0	0.0	1.3	11.0	44.6	20.2	22.9	43.1
**Mother’s occupation** (Mis a: 34,592; Mis b: 8,575)	Self-e/Enterpreneur		9,037	2,255 (25.0)	4.6 ± 1.4	0.0	0.9	11.7	46.4	24.3	16.7	41.0
	Manager		2,145	579 (27.0)	4.6 ± 1.4	0.0	1.2	12.0	47.1	23.9	15.8	39.7
	Employed-Clerk		31,002	7,213 (23.3)	4.7 ± 1.6	0.1	0.6	11.0	44.5	25.0	18.9	43.8
	Blue Collar		12,836	3,206 (25.0)	4.6 ± 1.5	0.0	0.4	12.8	43.6	28.5	14.6	43.1
	Other (employed)		19,634	4,639 (23.6)	4.7 ± 1.6	0.1	0.7	12.	43.9	24.6	18.4	42.9
**Father’s occupation** (Mis a: 10,867; Mis b: 2,935)	Self-e/Enterpreneur		22,100	5,171 (23.4)	4.6 ± 1.6	0.1	0.7	12.3	46.4	23.6	17.0	40.6
	Manager		3,338	965 (28.9)	4.6 ± 1.4	0.0	1.2	11.4	50.1	21.2	16.3	37.4
	Employed-Clerk		22,537	5,245 (23.3)	4.7 ± 1.6	0.1	0.6	11.7	46.0	22.5	19.1	41.7
	Blue Collar		32,812	7.988 (24.4)	4.7 ± 1.8	0.1	0.5	12.5	43.9	24.6	18.4	43.0
	Other (employed)		17,592	4,163 (23.7)	4.7 ± 1.7	0.2	0.7	13.6	43.9	22.0	19.6	41.6
**Consaguinity**	No		109,099	26,439 (24.2)	4.7 ± 1.7	0.1	0.6	11.9	44.5	24.8	18.1	42.9
	Yes		147	28 (19.1)	4.6 ± 1.3	0.0	3.6	14.3	25.0	42.9	14.3	57.1
**OBSTETRIC HISTORY FACTORS**												
**Previous** **Livebirths** (number)	0		58,217	14,523 (25.0)	4.8 ± 1.8	0.1	0.4	8.8	42.0	27.6	21.1	48.7
	1		39,805	9.265 (23.3)	4.5 ± 1.5	0.1	0.8	15.7	47.8	21.7	13.9	35.5
	2		8,644	2,137 (24.7)	4.5 ± 1.6	0.1	0.9	15.8	46.2	21.2	15.9	37.2
	3		1,820	411 (22.6)	4.7 ± 2.2	0.0	1.2	15.9	45.6	18.3	19.0	37.3
	4+		755	131 (17.4)	4.8 ± 2.3	0.0	2.3	12.3	47.7	16.9	20.8	37.7
**Previous stillbirths** (number)	0		108,502	26,137 (24.1)	4.7 ± 1.7	0.1	0.6	11.9	44.4	24.9	18.1	42.9
	1+		744	330 (44.4)	4.7 ± 1.6	0.6	0.3	8.8	48.5	21.0	20.7	41.8
**Previous** **cesarean sections** (number)	0		100,003	19,565 (19.6)	4.8 ± 1.7	0.1	0.6	9.9	42.0	26.7	20.7	47.4
	1		8,097	5,794 (71.6)	4.3 ± 1.4	0.0	0.6	17.5	51.6	19.8	10.5	30.3
	2+		1,146	1,108 (96/7)	4.4 ± 1.4	0.1	1.4	17.6	50.4	17.9	12.8	30.6
**Previous** **pre-term babies** (number) (Mis a:1,144; Mis b: 258)	0		105,774	25,365 (24.0)	4.7 ± 1.7	0.1	0.6	11.9	44.6	24.8	18.1	42.9
	1		2,041	717 (35.1)	4.7 ± 2.0	0.1	0.4	14.2	46.4	19.8	19.0	38.8
	2+		287	127 (44.3)	4.7 ± 1.9	0.0	0.8	18.4	43.2	16.8	20.8	37.6
**Previous** **pontaneous abortions** (number)	0		92,694	22,203 (24.5)	4.7 ± 1.7	0.1	0.6	11.5	45.2	24.9	17.8	42.7
	1		12,555	3,079 (6.0)	4.7 ± 1.6	0.1	0.7	15.0	40.1	23.9	20.3	44.2
	2		2,897	804 (27.8)	4.7 ± 1.6	0.1	0.6	12.3	41.5	27.2	18.3	45.5
	3+		1,099	381 (34.7)	4.7 ± 1.8	0.0	0.8	12.3	46.1	22.1	18.7	40.8
**Previous** **neonatal deaths** (number)	0		108,923	26,330 (24.2)	4.7 ± 1.7	0.1	0.6	11.9	44.5	24.8	18.1	42.9
	1+		323	137 (42.4)	4.9 ± 1.8	0.0	0.7	9.6	45.6	21.3	22.8	44.1

5 **Obstetric history** (Table 4): previous livebirths; previous CS; previous stillbirths; previous pre-term births; previous spontaneous abortions; previous neonatal deaths.

### Statistical analysis

The mean LoS and the percentage of LoS longer than the proposed ED benchmark (4 days) following CS were calculated for each of the above explanatory factors.

The 0/1 variable LoS (lower/higher than ED) was used as dichotomous outcome in a multiple logistic regression model.

The following factors were deliberately dropped from the final multivariate logistic regression model:

- Apgar score at 1 minute and resuscitation (because of collinearity with Apgar score at 5 minutes and ICU admission, both more plausible to be retained in the final model);
- child’s size (collinearity with birthweight and gestational age, both with stronger effect size than child’s size);
- father’s occupation and marital status (large number of missing values and relatively small effect size).

Sensitivity analysis was fitted by excluding marital status, pre-term history and father’s occupation from the final logistic regression model. Excluding the latter factors made little difference to the effect size estimates of any of the other factors (S1 Table).

Differences in mean LoS were also investigated with a multiple linear regression model including the same explanatory factors of the above multiple logistic regression analysis.

Only estimates for hospitals, calendar year (linear factor) and type of CS (planned vs. urgent) are displayed, reporting adjusted odds ratios (aOR) and adjusted regression coefficients (aRC) with 95% confidence intervals (95%CI) for each stratum specific compared to the reference. Hospital J was chosen as reference since it is the third maternity centre of FVG in terms of total number of births, had the second highest CS rate in the region and the shortest mean LoS post CS among all public hospitals.

Population attributable risks (PARs) were then calculated for each hospital in the ideal scenario of having the same performance as hospital J (reference) during calendar year 2015. For the calculation of PAR a function of Stata called “Regpar” was employed.

Additionally, the above calculation of PAR was also restricted to low risk pregnancies, defined as conditions of the mother and/or the newborn simultaneously meeting all the following criteria:

- Maternal age < 35 years;
- Mother without hypertension/diabetes;
- Singleton birth;
- Gestational age: 37–40 weeks;
- Birthweight: 2,500–3,999 g;
- Pre-delivery LoS ≤ 2 days;
- No labour induction;
- No administration of labour analgesia.
- Apgar score at 1 minute ≥7;
- Apgar score at 5 minute ≥8;
- No ICU admission;
- No resuscitation performed.

Missing values were excluded and complete case analysis was performed.

Stata 14.2 (College Station, Texas, USA) was employed for the analysis.

## Results

Fig 1 shows the flowchart displaying the various selection criteria applied to the initial eligible births (N = 109,246) to obtain the final number of CSs available for the analysis. In the entire FVG the total number of CSs was 26,467 during 2005–2015.

Table 1 shows the distribution of LoS (mean and proportion of LoS>ED) after CSs by calendar year (upper panel) and hospital (lower panel). The pooled mean LoS in FVG during 2005–2015 equaled 4.7 days and it consistently exceeded our proposed ED benchmark for CS in all hospitals. Although there was a decreasing trend (p<0.001) over the years in the mean LoS (5.0 ± 1.7 days in 2005 vs. 4.4 ± 1.7 days in 2015) and in the percentage of LoS > ED (58.2% in 2005 vs. 27.9% in 2015), the variability among the 12 maternity centres was considerable. The mean LoS ranged from 4.2 days (hospital H) up to 5.5 days (hospital D), whereas the percentage of LoS > ED ranged from 13.5% (hospital C) up to 87.3% (hospital D). A high percentage of LoS>ED was generally accompanied by greater mean LoS in individual maternity centres. Notably, despite having a mean LoS similar to other hospitals, centre C had a remarkably lower percentage of Los >ED (13.5%).

Table 2 shows the distribution of LoS by maternal health factors. LoS post CS was higher for pre-delivery LoS>6 days (5.7 ± 2.9 days), hypertension/diabetes (5.6 ± 2.6 days), stillbirth (5.4 ± 3.5 days, shoulder presentation (5.3 ± 2.4 days), maternal age higher than 45 years (5.3 ± 2.6 days), pre-delivery LoS 3–5 days (5.1 ± 2.2) and <4 obstetric checks during pregnancy (5.0 ± 1.8), The highest proportion of LoS>ED corresponded to mother’s age >45 (60.9%), hypertension/diabetes (63.8%), pre-delivery LoS >6 days (61.6%), shoulder presentation (59.2%) and < 4 midwife checks received during pregnancy (53.1%).

Table 3 displays the distribution of LoS by clinical factors of the child. The main child size factors associated with higher mean LoS were gestational age less than 29 weeks (5.7 ± 3.1 days), low birthweight (birthweight = 2,000–2,499 g; 5.5 ± 2.2 days), gestation of 33–36 weeks (5.5 ± 2.3 days), birthweight<2,000 g (5.4 ± 2.6), placenta weighing more than 1Kg (5.3 ± 2.1 days) and gestational age of 5.2 ± 2.4 days. Regarding child’s fragility factors, the higher mean LoS was found for multiple births (5.2 ± 1.9 days), ICU admission (5.4 ± 2.5 days), resuscitation (5.4 ± 2.7 days), Apgar score at 5 minutes less than 8 (5.3 ± 2.6) and Apgar score at 1 minute less than 7 (5.2 ± 2.5 days). The same patterns were observed for the proportions of LoS >ED.

Table 4 shows the distribution of LoS by socio-demographic and obstetric history factors. A higher mean LoS was found with father’s age of 15–19 years (5.1 ± 1.8 days), father’s age 50–54 years (4.9 ± 1.9), lower maternal education (5.0 ± 2.0), lower paternal education (4.9 ± 2.0) and history of neonatal death (4.9 ± 1.8). The highest proportion of LoS >ED was found for consanguinity of parents (57.1%). The mean LoS as well as the percentage of LoS>ED slightly increased with decreasing educational level of both parents and decreased with higher number of previous CS.

**Fig 3:**
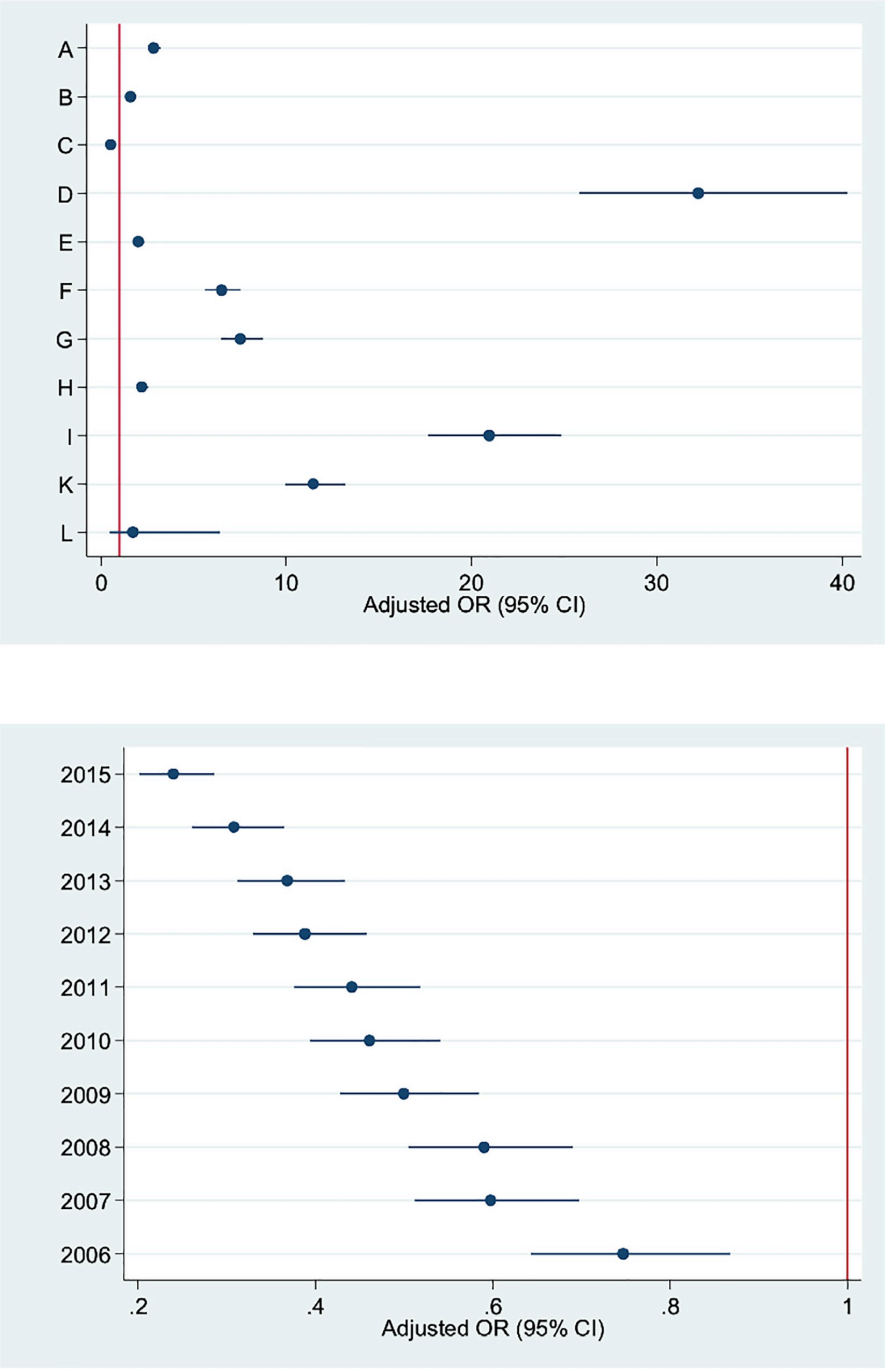
Scatter plot displaying the adjusted odds ratios (OR) with 95% confidence intervals (95%CI) of LoS surpassing the ED (4 days) for caesarean sections, by facility centre and calendar year.

Table 5 shows the results of the final multivariable logistic regression model in the whole FVG. Only aORs related with calendar year (linear term), hospital and type of CS are shown. A significantly decreasing time trend of LoS >ED was observed (aOR = 0.88; 95%CI: 0.82; 0.94). With the exception of hospital C (aOR = 0.55; 95%CI: 0.47; 0.66), all other maternity centres were by far more likely to keep women admitted more than four days as compared to J (the reference). The probability of LoS being longer than ED ranged from 1.64 (95%CI: 1.47; 1.83) in hospital B up to 32.04 (95%CI: 25.62; 40.06) in hospital D. Fig 3 displays the scatter plot of the adjusted ORs by facility centre (upper graph) and calendar year (lower graph).

**Table 5:** Multivariable logistic and linear regression model on length of stay (LoS, in days) following cesarean section (CS). 24,729 (complete case) observations. Adjusted regression coefficients (aRC)^#^ and adjusted odds ratios (aOR)^**$**^, with 95% confidence interval (95%CI); population attributable risk (PAR-1* and PAR-2**) with 95%CI.

FACTORS		Mean LoS	LoS >4 days vs. ≤ 4 days		
		aRC (95%CI)	aOR (95%CI)	PAR-1 (95% CI)*	PAR-2 (95% CI)**
**Hospital**	A	0.19 (0.17; 0.21)	2.85 (2.54; 3.20)	+27.8% (+25.8%; +29.7%)	+21.7% (+18.7%; +24.7%)
	B	0.08 (0.06; 0.10)	1.64 (1.47; 1.83)	+16.8% (+15.2%; +18.5%)	+12.3% (+10.2%; +14.3%)
	C	-0.07 (-0.10; -0.05)	0.55 (0.47; 0.66)	+1.0% (-1.4%; +2.7%)	+0.6% (-0.7%; +1.9%)
	D	0.64 (0.61; 0.67)	32.04 (25.62; 40.06)	+72.1% (+69.5%; +74.5%)	+72.3% (+68.5%; +75.7%)
	E	0.12 (0.09; 0.14)	1.99 (1.71; 2.32)	+20.5% (+17.8%; +23.2%)	+15.3% (+12.4%; +18.2%)
	F	0.38 (0.35; 0.40)	6.60 (5.70; 7.63)	+45.8% (+43.1%; +48.4%)	+39.9% (+35.5%; +44.1%)
	G	0.40 (0.37; 0.43)	7.48 (6.44; 8.68)	+48.4% (+45.7%; +51.1%)	+42.8% (+38.4; +47.0%)
	H	0.14 (0.11; 0.16)	2.21 (1.93; 2.54)	+22.6% (+20.2%; +25.0%)	+17.1% (+14.2%; +8.8%)
	I	0.59 (0.56; 0.62)	20.83 (17.54; 24.75)	+66.7% (+64.2%; +69.0%)	+64.9% (+61.2%; +68.3%)
	J	*reference*	*reference*	*reference*	*reference*
	K	0.49 (0.47; 0.52)	11.81 (10.23; 13.63)	+57.4% (+55.0%; +59.7%)	+53.2% (+49.1%;+ 57.1%)
	L	0.12 (-0.14; 0.37)	1.97 (0.51; 7.65)	NA	NA
**Calendar year (2005–2015)**		-0.021 (-0.022; -0.019)	0.89 (0.88; 0.90)		
**Cesarean type**	Urgent	reference	reference		
	Planned	-0.02 (-0.04; -0.01)	0.88 (0.82; 0.94)		

**#,$** Multiple linear and logistic regression models, both adjusted for the following domain factors (displayed in Tables 1–4):

- **Health care setting and timeframe** (hospital; calendar year)

- **Maternal health factors** (type of cesarean section; maternal age; hypertension/diabetes; amniocentesis; number of obstetric checks; number. of US scans performed; induction mode; labour analgesia; neonatal status; presentation; pre-delivery LoS)

- **Child’s fragility factors** (Apgar score at 5 minutes; ICU admission; multiple birth)

- **Child’s size factors** (gestational age; birthweight; placenta weight)

- **Obstetric history factors** (parity; history of cesarean sections, pre-term history)

- **Socio-demographic factors** (paternal age; mother’s nationality; mother’s education)

* **Population Attributable Risk 1 (PAR-1):** Proportional variation of LoS < ED after childbirth in the ideal scenario each hospital would be performing as the reference (hospital J) during calendar year 2015.

** Population Attributable Risk 2 (PAR-2): Proportional variation of LoS < ED after childbirth in the ideal scenario each hospital would be performing as hospital J during calendar year 2015. Estimates of PAR-2 calculated only for low risk pregnancies, defined as conditions of the mother and/or the newborn simultaneously meeting all the following criteria: mother’s age<35; no women affected by hypertension/diabetes; gestational age: 37–40 weeks; singleton birth; pre delivery LoS ≤ 2 days; no labour induction; no administration of labour analgesia; no resuscitation performed; child not admitted to ICU; Apgar score at 1 minute ≥7; Apgar score at 5 minutes ≥8; birthweight: 2,500–3,999gr.

In the ideal scenario each hospital would be performing as hospital J during calendar year 2015 a variable and strong increase of ED rate would be observed for all centres but C. In particular, the proportional increase of ED in this fantasy scenario would range from +16.8% (centre B) up to +72.1% (centre D), and it would reach significantly high proportions in hospital I (+66.7%), K (+57.4%), G (+48.4%), F (+45.8%), A (+27.8%), H (+22.6%) and E (+20.5%) in descending order (Table 5). The same pattern of PAR was confirmed for low risk pregnancies (PAR 2), which were 205 out of all CSs performed in FVG during 2005–2015.

Table 5 also shows the output of the multiple linear regression analysis. Only centre C (aRC = -0.07, 95%CI: -0.10; -0.05) had a mean LoS significantly lower than the reference; hospital D (aRC = 0.64; 95%CI: 0.61; 0.67), I (aRC = 0.59; 95%CI: 0.56; 0.62), K (aRC = 0.49; 95%CI: 0.47; 0.52) and G (aRC = 0.40; 95%CI: 0.37; 0.43) had the highest mean LoS among all maternity centres. The differences in aRCs consistently followed the differences in aORs.

## Discussion

### Key findings

In the whole FVG during 2005–2015 the pooled mean LoS post CS was 4.7 days, hence higher than the respective international ED benchmark, and the pooled proportion of LoS>ED was 42.9%. Regardless it was expressed as aRC or aOR, LoS post-CS was lowest in hospital C, highest in hospital D and intermediate in centres I, K, G, F, A, H, E, B and J (in descending order). The decreasing trend of LoS over the years in the whole FVG was rather negligible as compared to the differences observed among the various regional maternity centres. Unadjusted proportions of LoS >ED were similar to aORs and aRCs in ranking hospitals of FVG by LoS. If all other FVG hospitals were performing as hospital J during calendar year 2015 a variable and strong proportional increase of ED would be observed for all centres but C. Lastly, planned CSs had a significantly smaller mean LoS and were less likely to have a LoS >ED as compared to urgent/emergency CSs in the whole region during 2005–2015.

### Strengths and limitations

Besides being a population-based study, our investigation has several other strengths:

- the database is highly reliable since data were collected by trained health care staff;
- the proportion of missing values was negligible (mainly related to socio-demographic information);
- the large number of records allowed for substantial statistical power and accuracy of results.

Clinical and personal information of women and newborns that we had from the regional repository of FVG included more or less the same information of the Robson classification system, proposed by WHO in 2015 as a global standard for assessing, monitoring and comparing CS rates by setting and time [25]. In agreement with the WHO statement [25], which recommends monitoring CS rates “*at hospital level*” and “*in a standardized and action-oriented manner with the inclusion of maternal and perinatal outcomes*”, our analysis took into account the effect of a considerable number of factors that may affect LoS, and compared hospital performance by multivariable linear as well as logistic regression models to adjust the respective results for potential confounders. Since it is sound, the analytical methodology of the present study to assess and compare LoS may be generalized to other Italian regions and/or countries with health systems comparable to FVG. The use of the Robson classification system has been increasingly applied worldwide over the last decade and new studies to monitor and contrast CS rates are expected to be released in the near future. Likewise, it would be also recommended to compare LoS post CS by setting and time using a systematic approach as we did in the present study, taking into account the effect to multiple diverse factors [28–29].

A limitation of the present study is the lack of information on lifestyle habits (smoking, physical exercise, BMI), all reportedly being relevant determinants of CS and LoS post CS [30–34].

### Interpretation of findings

The mean LoS after CS and the proportion of LoS >4 days, both adjusted for all potential determinants, achieved the least values in hospital C, the only private maternity centre of FVG. Therefore, the target of ED (4 days) after CS may be feasible from an organizational and budgeting perspective. Although being a private facility, the health care costs for patients in centre C are the same as in public hospitals, because the former is funded by the regional government conditional on a temporary contract. The latter convention needs to be renewed upon expiry, subject to evaluation of health outcomes delivery, management controls and audits on performance efficiency. The other 11 public hospitals behaved less efficiently than C, regardless of their rank in terms of specialization level and size of the population served [35]. In fact, the variability of practice pattern among maternity centres was confirmed also in low risk pregnancies; moreover adjusted and unadjusted proportions of LoS >ED were rather similar, suggesting a similar case-mix of hospitals.

The standardization of practice patterns at hospital level requires pro-active interventions, because the “spontaneous” improvements of LoS post CS over time observed during these 11 years were rather marginal as compared to the variability across the various maternity centres.

### Generalizability

#### Worldwide figures on LoS post CS

Worldwide national administrative figures on LoS post CS are scarce or missing.

Considering countries/regions with universal health coverage as Italy, FVG performed much better than South Korea, which reportedly had an average LoS of 6.5 days following 150,256 CSs during 2012–14 [36]. However in Sweden, another country whose health system is funded by central taxation as Italy’s, the average LoS was 2.2 days following VD and 3.7 days post CS for women delivering in hospitals during 2009 [37]. Similarly, in New South Wales (Australia) the overall mean LoS after CS declined from 3.7 days in 2001 up to 3.4 days in 2007. Although the mean LoS reduced both for VD and CS and for both private and public hospitals in New South Wales, private facilities showed longer LoS following CS as well as VD [38]. By contrast, in our study the only private maternity centre of FVG had shorter LoS post CS than public hospitals. Likewise, the median LoS was 3 days among 57,067 women delivering by CS between 1999 and 2002 in 19 academic hospitals in the US, a country whose health system is funded by private voluntary health insurance [14,39–42]. Data from nationally representative surveys in the US reported a considerably decreasing trend over time in the mean LoS following CS, from 7.9 days in 1970, to 6.5 days in 1980, 4.0 days in 1992 and 3.6 days in 2006. The reduction of LoS over the years was much more pronounced post CS than VD (3.2 days in 1980 vs. 2.2 days in 2006) [14, 40–42].

Some central European countries with health systems funded by social insurances have also been increasingly applying ED policies. For instance, in France, (although with a relaxed ED definition: <3 days for VD and <5 days for CS) post-partum ED concerned 3% out of all births in 1997 vs. 7% in 2002. Focusing on medically fit parturients, it is estimated that 40% VDs and 25% CSs among primiparas and 55% VDs and 30% CSs among multiparas could be discharged “early” in France [43].

#### Evidence supporting ED policies

In several European and high-income countries elsewhere free access to maternity health-care is provided by universal health coverage funded by central taxation [39]. In light of this, policy makers have been trying to contract unnecessary extended LoS to pursue a cost-effective management of health care resources whilst maintaining quality of care [44]. Nevertheless, this approach has been implemented also by countries with different health systems.

LoS was 77% longer and childbirth 76% more costly for planned CS as compared to planned VD in a study conducted on 244,088 women from Massachusetts (US) [45]. Overlapping figures were reported from 30,168 obstetric records (10,897 VD and 19,271 CS), drawn from 18 tertiary hospitals of Chongqing Municipality in China during 2011–2013, when LoS after CS was 77% longer than that of a VD, with an average hospital cost being 76% higher than the average cost for a VD [46].

Although re-hospitalization rates could not be inversely related with LoS [45], reduction of hospitalization length may considerably shorten the observation time of patients to detect latent signs of disease and provide recommendations for newborn care, thus increasing the risk of readmission. However, as can be seen below, the evidence of the impact of ED on maternal and newborn health is still inconclusive both for CS and VD [13,17,38,47–50].

Among 102 women undergoing CS at Pennsylvania University Hospital (US) between 1988 and 1991, 61 were randomized to early discharge, 61 to receive standard care (controls). Women undergoing ED accompanied by transitional home care provided by clinical nurse specialists were discharged on average 30.3h earlier than controls (p <0.001). Women going through ED resulted to be significantly more satisfied with the health care received, were charged 29% less than the control group and their children received more timely immunizations. Furthermore, no difference in maternal and newborn health outcomes were found. Whilst more maternal readmissions occurred in the control than in the ED group (3 vs. 0), numbers involved were in fact too small to account for statistical difference [22].

A study was conducted in Iceland during 2008–2009 to test a fast-track discharge program (defined as ≤ 48 hours) on 213 women (182 fast-track) following singleton birth by planned CS. LoS by parity in the latter group of women was compared with 199 women delivering by planned CS in 2003 and 183 delivering in 2007. The median LoS decreased from 81 hours in 2007 to 52 hours in 2008–9, when 66% women were discharged within 48 hours post CS. BMI and parity had negligible impact on LoS during 2008–9 in the latter study, although nulliparous parturients aged ≤ 25 years were more likely to stay more than 48 h. The number of re-hospitalizations was equal to 4 in each period and women in the fast-track program were reportedly satisfied with ED. Therefore, most healthy women could be discharged within 48 hours following planned CS for singleton pregnancy, with little or no risk of readmissions [51].

Canada is a country particularly active in implementing and evaluating ED policies. In a population-based study conducted in Canada from 1989 to 1999 on 2,652,726 parturients, the mean LoS post low-risk planned CS was 3.96 days versus 2.56 days following low risk VD. Those delivering by CS were reportedly more likely to be readmitted to hospital in the first week after discharge than those undergoing VD (53% vs 41%). Among women delivering by CS, LoS ≤2, 3, and 4 days were associated with 21%, 18%, and 10% higher risk of re-admission (estimates adjusted only for maternal age) respectively, as compared to women with LoS equal to 5 days. The authors concluded that short LoS following CS may increase the risk of readmission [13]. Since readmissions are costly, the contraction of LoS could therefore not necessarily translate into efficient use of resources [13,52]. The latter was a population-based study, with estimates controlled only for the effect of maternal age; however, variability of LoS at hospital level can be driven by various factors [44]. In fact, in a more recent population-based study conducted in Quebec (Canada) on 1,875,322 livebirths, LoS peaked at day 1 (47.3%) following VD and at day 3 (49.3%) post CS. Readmission rates were 4.2% for VD and 2.2% after CS. The authors concluded that neonatal readmission rates in the country were not increasingly attributable to diminishing LoS, they could rather be explained by changes in the day-specific readmission rates. Readmission rates were lowest for LoS of 1–2 days post VD and for LoS of 2–4-days following CS if outpatient community care was provided [52].

In order to recognize the characteristics of mothers and/or newborns needing to remain hospitalized longer to avoid potential subsequent re-admissions following ED for CS, it would be interesting to assess the effect of LoS and the eventual introduction of ED policies on re-hospitalization rates, using a case-mix approach as we did in the present study.

### Prospects

In a previous study in FVG during the same timeframe, we found an overall mean LoS of 2.9 days following spontaneous vaginal deliveries (SVD) and 3.3 days after instrumental vaginal deliveries (IVD) [17]. Differences in LoS post CS by maternity centres were milder than LoS after SVD in the latter study. Whilst some FVG hospitals showed a relatively consistent pattern of LoS by delivery mode, some other completely reversed their behaviour between SVD/IVD and CS, especially the two regional referral hospitals (centres A and B). This may be attributable to a rather different approach of maternity centres in the management of postnatal care, depending on the delivery mode.

It is estimated that up to 50% of the reasons women do not need to remain admitted after childbirth are under direct control of the hospital itself and often relate to internal decision-making and/or organizational malfunctions, such as [15–17,34,36]:

- economic convenience;
- inefficiency of hospital processes (resulting in patient treatment being delayed);
- medical errors and low quality of care (determining need of further treatment and longer recovery time);
- insufficient coordination among different services within the health system (resulting in patients remaining bogged down in hospitals, waiting for future care to be planned);
- fear of medico-legal consequences.

Hospitals are in the position to control medical costs, shorten average LoS and accelerate the bed turnover whilst guaranteeing quality of care [46]. However, trying to contain unnecessary extended LoS is a difficult task requiring considerable effort [44,53]. A number of principles of good clinical practice have been already proposed [15]. One of the most important of these points is education of health care staff and society [15]. In this respect, stewardship programs have proven to be effective in reducing LoS [54]. Another crucial point is the devolution of responsibility to ward staff, with allowance to try and test ideas/changes as part of their daily activities, following a “bottom-up” approach [15].

In our study LoS post planned CS was less likely to surpass the ED benchmark when compared to urgent/emergency CS. As far as we are aware this finding has never been reported in the open literature. Many obstetric units in the UK have either introduced or are planning to introduce enhanced recovery (ER) as a means to reduce LoS after planned CS. The aim of ER is to optimize multiple aspects of patient care and improve recovery, thereby facilitating ED whilst maintaining quality of care and patient satisfaction [4,55]. The implementation of ER programs post planned CS results in multiple advantages: reduction of LoS, reduction of morbidity and earlier return home to normal life for women [4,55–57].

## Conclusions

The mean LoS post CS in FVG during 2005–2015 FVG was 4.7 days, hence higher than the international threshold recommended post CS. Although there was a significantly decreasing trend in the average LoS and proportion of LoS>ED over the years in the whole region, considerable variability was observed by maternity centre. FVG hospitals have the power to improve internal organizational processes to shorten LoS and accelerate the bed turnover whilst guaranteeing quality of care. Policy makers could effectively improve the management and clinical governance of maternity services by enforcing standardized guidelines, using audits, economic incentives and penalties if need be. For any case of LoS>ED following CS medical records should be scrutinized to justify prolonged LoS. However, integration of inpatient and outpatient services is a critical step to ensure that the mother and the newborn receive appropriate follow-up care in the community [52]. Moreover, re-admission rates should be investigated in relation to the eventual introduction of ED policies post CS, using a systematic approach, also employing patients’ satisfaction surveys.

## Supporting information

S1 Fig Questionnaire. Certificate of Delivery Care (CEDAP).
(DOC)

S1 Table Sensitivity analysis. Logistic regression models.
(DOCX)
