# Correction: Length of stay following cesarean sections: A population based study in the Friuli Venezia Giulia region (North-Eastern Italy), 2005-2015

**DOI:** 10.1371/journal.pone.0213939

**Published:** 2019-03-12

**Authors:** Luca Cegolon, Giuseppe Mastrangelo, Oona M. Campbell, Manuela Giangreco, Salvatore Alberico, Lorenzo Monasta, Luca Ronfani, Fabio Barbone

The sixth author’s name is spelled incorrectly. The correct name is: Lorenzo Monasta. The correct citation is: Cegolon L, Mastrangelo G, Campbell OM, Giangreco M, Alberico S, Monasta L, et al. (2019) Length of stay following cesarean sections: A population based study in the Friuli Venezia Giulia region (North-Eastern Italy), 2005–2015. PLoS ONE 14(2): e0210753. https://doi.org/10.1371/journal.pone.0210753

In the Study Design subsection of the Methods, there is an error in the final sentence of the paragraph. The correct sentence is: Data analyzed in this study were fully anonymized before being accessed, hence informed consent from patients was waived from the Regional Health Authority of FVG.

In the fourth paragraph of the Results, there is an error in the second sentence. The correct sentence is: The main child size factors associated with higher mean LoS were gestational age less than 29 weeks (5.7 ± 3.1 days), low birthweight (birthweight = 2,000–2,499 g; 5.5 ± 2.2 days), gestation of 33–36 weeks (5.5 ± 2.3 days), birthweight < 2,000 g (5.4 ± 2.6), placenta weighing more than 1Kg (5.3 ± 2.1 days) and gestational age of 29–32 weeks (5.2 ± 2.4 days).

There is a typographical error in the Factors column in Table 4. Please see the correct Table 4 here.

**Table 4:** Distribution of length of stay (LoS, in days) after cesarean section (CS) by socio demographic and obstetric history factors. Number (N), percentage (%); mean LoS ± standard deviation (SD); Mis a: missing values on all births; Mis b: missing values considering only CS. Self-e = self-employed.

FACTORS	STRATA		ALL BIRTHS (N = 109,246)	CESAREAN SECTIONS (N = 26,467)								
				Number (Row %)	LoS (days)							
					Mean ± SD	≤1	2	3	4	5	6+	>4
						Row %						
**SOCIO-DEMOGRAPHIC FACTORS**												
**Father’s age** (years) (Mis a: 1,949; Mis b: 495)	15–19		199	20 (10.1)	5.1 ± 1.8	0.0	0.0	10.0	35.0	35.0	20.0	55.0
	20–24		2,798	480 (17.2)	4.7 ± 1.7	0.0	0.2	14.3	42.2	24.8	18.5	43.3
	25–29		12,982	2,696 (20.8)	4.7 ± 1.8	0.1	0.7	12.1	45.5	23.9	17.7	41.6
	30–34		31,601	7,168 (22.7	4.6 ± 1.6	0.1	0.9	13.0	44.6	25.2	16.2	41.4
	35–39		34,560	8,478 (24.5)	4.7 ± 1.6	0.1	0.5	11.9	44.5	25.1	17.9	43.0
	40–44		17,866	4,898 (27.4)	4.7 ± 1.7	0.1	0.6	11.2	44.3	24.6	19.3	43.9
	45–49		5,353	1,632 (30.5)	4.7 ± 1.5	0.0	0.5	11.3	44.6	23.7	20.0	43.7
	50–54		1,361	420 (30.9)	4.9 ± 1.9	0.0	8.0	8.0	43.0	26.5	21.6	48.1
	55+		577	180 (31.2)	4.8 ± 1.6	0.0	1.1	7.8	45.6	25.6	20.0	45.6
**Mother’s** **nationality** (Mis a:116; Mis b: 36)	EU	Italian	86,083	20,662 (24.0)	4.6 ±1.6	0.1	0.6	12.0	44.7	25.2	17.4	42.6
		Non-Italian	5,983	1,242 (20.8)	4.4 ±1.3	0.2	0.9	14.2	49.0	23.4	12.4	35.8
	Non-EU		17,064	4,527 (26.5)	4.9 ± 2.1	0.1	0.5	10.8	42.4	23.2	23.0	46.2
**Marital status** (Mis a: 8,155; Mis b: 2,068)	Not married		12,036	2,872 (23.9)	4.8 ± 1.8	0.1	0.7	8.7	44.0	24.7	21.8	46.5
	Married		70,340	17,136 (24.4)	4.7 ± 1.7	0.1	0.6	12.6	43.5	24.7	18.5	43.2
	Separated		1,136	606 (32.1)	4.7 ± 2.1	0.2	0.3	13.0	43.2	23.1	20.1	43.2
	Widow		82									
	Divorced		669									
	Living together		16,846	3,785 (22.5)	4.6 ± 1.6	0.0	0.8	13.7	45.4	24.2	16.0	40.2
**Mother’s education** (Mis a: 24; Mis b: 9)	University or more		29,150	6,935 (23.8)	4.7 ± 1.6	0.1	0.7	11.5	45.9	23.0	18.9	41.9
	Secondary		52,988	12,617 (23.8)	4.6 ± 1.6	0.1	0.6	12.4	44.3	25.6	17.1	42.7
	Junior Secondary		25,107	6,347 (25.3)	4.7 ± 1.9	0.1	0.6	11.8	43.4	25.3	18.7	44.0
	Primary/none		1,977	559 (28.3)	5.0 ± 2.0	0.0	0.9	7.8	43.2	22.9	25.2	48.1
**Father’s education** (Mis a: 6,772; Mis b: 1,798)	University or more		18,542	4,527 (24.4)	4.6 ± 1.6	0.1	0.8	12.1	46.3	21.5	19.2	40.7
	Secondary		51,356	12,156 (23.7)	4.6 ± 1.6	0.1	0.5	13.1	44.9	23.9	17.5	41.4
	Junior Secondary		30,767	7,510 (24.4)	4.7 ± 1.8	0.1	0.7	11.2	45.3	23.6	19.1	42.8
	Primary/none		1,809	476 (26.3)	4.9 ± 2.0	0.0	1.3	11.0	44.6	20.2	22.9	43.1
**Mother’s occupation** (Mis a: 34,592; Mis b: 8,575)	Self-e/Enterpreneur		9,037	2,255 (25.0)	4.6 ± 1.4	0.0	0.9	11.7	46.4	24.3	16.7	41.0
	Manager		2,145	579 (27.0)	4.6 ± 1.4	0.0	1.2	12.0	47.1	23.9	15.8	39.7
	Employed-Clerk		31,002	7,213 (23.3)	4.7 ± 1.6	0.1	0.6	11.0	44.5	25.0	18.9	43.8
	Blue Collar		12,836	3,206 (25.0)	4.6 ± 1.5	0.0	0.4	12.8	43.6	28.5	14.6	43.1
	Other (employed)		19,634	4,639 (23.6)	4.7 ± 1.6	0.1	0.7	12.	43.9	24.6	18.4	42.9
**Father’s occupation** (Mis a: 10,867; Mis b: 2,935)	Self-e/Enterpreneur		22,100	5,171 (23.4)	4.6 ± 1.6	0.1	0.7	12.3	46.4	23.6	17.0	40.6
	Manager		3,338	965 (28.9)	4.6 ± 1.4	0.0	1.2	11.4	50.1	21.2	16.3	37.4
	Employed-Clerk		22,537	5,245 (23.3)	4.7 ± 1.6	0.1	0.6	11.7	46.0	22.5	19.1	41.7
	Blue Collar		32,812	7.988 (24.4)	4.7 ± 1.8	0.1	0.5	12.5	43.9	24.6	18.4	43.0
	Other (employed)		17,592	4,163 (23.7)	4.7 ± 1.7	0.2	0.7	13.6	43.9	22.0	19.6	41.6
**Consaguinity**	No		109,099	26,439 (24.2)	4.7 ± 1.7	0.1	0.6	11.9	44.5	24.8	18.1	42.9
	Yes		147	28 (19.1)	4.6 ± 1.3	0.0	3.6	14.3	25.0	42.9	14.3	57.1
**OBSTETRIC HISTORY FACTORS**												
**Previous** **Livebirths** (number)	0		58,217	14,523 (25.0)	4.8 ± 1.8	0.1	0.4	8.8	42.0	27.6	21.1	48.7
	1	39,805	9.265 (23.3)	4.5 ± 1.5	0.1	0.8	15.7	47.8	21.7	13.9	35.5
	2	8,644	2,137 (24.7)	4.5 ± 1.6	0.1	0.9	15.8	46.2	21.2	15.9	37.2
	3	1,820	411 (22.6)	4.7 ± 2.2	0.0	1.2	15.9	45.6	18.3	19.0	37.3
	4+	755	131 (17.4)	4.8 ± 2.3	0.0	2.3	12.3	47.7	16.9	20.8	37.7
**Previous stillbirths** (number)	0		108,502	26,137 (24.1)	4.7 ± 1.7	0.1	0.6	11.9	44.4	24.9	18.1	42.9
	1+	744	330 (44.4)	4.7 ± 1.6	0.6	0.3	8.8	48.5	21.0	20.7	41.8
**Previous** **cesarean sections** (number)	0		100,003	19,565 (19.6)	4.8 ± 1.7	0.1	0.6	9.9	42.0	26.7	20.7	47.4
	1	8,097	5,794 (71.6)	4.3 ± 1.4	0.0	0.6	17.5	51.6	19.8	10.5	30.3
	2+	1,146	1,108 (96/7)	4.4 ± 1.4	0.1	1.4	17.6	50.4	17.9	12.8	30.6
**Previous** **pre-term babies** (number) (Mis a:1,144; Mis b: 258)	0		105,774	25,365 (24.0)	4.7 ± 1.7	0.1	0.6	11.9	44.6	24.8	18.1	42.9
	1	2,041	717 (35.1)	4.7 ± 2.0	0.1	0.4	14.2	46.4	19.8	19.0	38.8
	2+	287	127 (44.3)	4.7 ± 1.9	0.0	0.8	18.4	43.2	16.8	20.8	37.6
**Previous** **spontaneous abortions** (number)	0		92,694	22,203 (24.5)	4.7 ± 1.7	0.1	0.6	11.5	45.2	24.9	17.8	42.7
	1	12,555	3,079 (6.0)	4.7 ± 1.6	0.1	0.7	15.0	40.1	23.9	20.3	44.2
	2	2,897	804 (27.8)	4.7 ± 1.6	0.1	0.6	12.3	41.5	27.2	18.3	45.5
	3+	1,099	381 (34.7)	4.7 ± 1.8	0.0	0.8	12.3	46.1	22.1	18.7	40.8
**Previous** **neonatal deaths** (number)	0		108,923	26,330 (24.2)	4.7 ± 1.7	0.1	0.6	11.9	44.5	24.8	18.1	42.9
	1+	323	137 (42.4)	4.9 ± 1.8	0.0	0.7	9.6	45.6	21.3	22.8	44.1
