# Supplementary material for: Length of stay following cesarean sections: A population based study in the Friuli Venezia Giulia region (North-Eastern Italy), 2005-2015
Source: PLoS One. 2019 Feb 27;14(2):e0210753. doi: 10.1371/journal.pone.0210753 (PMC6392330; doi:10.1371/journal.pone.0210753)
Supplement: S1 Table — Logistic regression models. (DOCX) [file pone.0210753.s002.docx]

| **S2 Table (Sensitivity analysis).** Multiple logistic regression analysis adjusted for hospital, calendar year, type of cesarean section (CS) and all other significant terms from tables 1-4, included in the final multivariable logistic regression model whose results are showed in Table 5. Adjusted Odds ratios (aOR) with 95% confidence interval (95%CI); LoS=length of hospital stay (in days). | | | | | | | |
| --- | --- | --- | --- | --- | --- | --- | --- |
| **FACTORS** | | **STRATA** | **CESAREAN SECTIONS**  **aOR* (95%CI)**  **(LoS >4 vs. ≤ 4)** | | | | |
|  |  |  | **Final model ***  (24,670 observations) | **Adding father’s occupation**  (22,366 obs.) | **Adding pre-term history**  (24,427 obs.) | **Adding marital status**  (22,746 obs.) |  |
| **Type of**  **Cesarean section** | | Emergency | reference | reference | reference | reference |  |
|  |  | Planned | 0.88 (0.82; 0.94) | 0.87 (0.81; 0.94) | 0.88 (0.83; 0.94) | 0.88 (0.82; 0.94) |  |
| **HOSPITAL** | | A | 2.85 (2.54; 3.20) | 2.89 (2.57; 3.26) | 2.84 (2.53; 3.19) | 2.86 (2.54; 3.22) |  |
|  |  | B | 1.64 (1.47; 1.83) | 1.65 (1.47; 1.85) | 1.64 (1.47; 1.83) | 1.65 (1.47; 1.84) |  |
|  |  | C | 0.55 (0.47; 0.66) | 0.57 (0.48; 0.68) | 0.55 (0.47; 0.66) | 0.56 (0.47; 0.67) |  |
|  |  | D | 32.03 (25.62; 40.06) | 33.91 (26.93; 42.70) | 32.00 (25.59; 40.02) | 31.90 (25.21; 40.36) |  |
|  |  | E | 1.99 (1.71; 2.32) | 1.95 (1.67; 2.27) | 1.97 (1.70; 2.30) | 1.92 (1.64; 2.25) |  |
|  |  | F | 6.60 (5.70; 7.63) | 3.62 (2.71; 4.84) | 6.59 (5.70; 7.63) | 6.69 (5.75; 7.78) |  |
|  |  | G | 7.48 (6.44; 8.68) | 7.67 (6.58; 8.94) | 7.48 (6.44; 8.69) | 7.65 (6.52; 8.97) |  |
|  |  | H | 2.21 (1.93; 2.54) | 2.26 (1.96; 2.60) | 2.21 (1.93; 2.54) | 2.30 (2.00; 2.65) |  |
|  |  | I | 20.83 (17.54; 24.75) | 21.20 (17.77; 25.28) | 20.37 (16.97; 24.45) | 22.77 (18.80; 25.57) |  |
|  |  | J | reference | reference | reference | Reference |  |
|  |  | K | 11.81 (10.23; 13.63) | 11.85 (10.23; 13.72) | 11.81 (10.23; 13.63) | 11.83 (10.23; 13.68) |  |
|  |  | L | 1.97 (0.51; 7.65) | 2.00 (0.47; 6.48) | 1.93 (0.50; 7.45) | 1.98 (0.51; 7.68) |  |
| **Calendar year (2005-2015)** | | | 0.88 (0.88; 0.94) | 0.89 (0.88; 0.90) | 0.89 (0.88; 0.90) | 0.89 (0.88; 0.90) |  |
| **Father**  **occupation** | Self-e/Enterpreneur | |  | reference |  |  |  |
|  | Manager | |  | 0.96 (0.81; 1.13) |  |  |  |
|  | Employed-Clerk | |  | 1.10 (1.00; 1.21) |  |  |  |
|  | Blue Collar | |  | 1.19 (1.08; 1.30) |  |  |  |
|  | Other (employed) | |  | 1.02 (0.92; 1.13) |  |  |  |
| **Pre-term histotry**  (N. children) | 0 | |  |  | reference |  |  |
|  | 1 | |  |  | 1.14 (0.94; 1.37) |  |  |
|  | 2+ | |  |  | 1.10 (0.71; 1.72) |  |  |
| **Marital status** | Married | |  |  |  | reference |  |
|  | Non-married | |  |  |  | 0.93 (0.84; 1.03) |  |
|  | Widow/separated/divorced | |  |  |  | 0.89 (0.72; 1.10) |  |

* Multiple logistic regression model adjusted for the following domain factors displayed in table 1- 4

- **Health care setting and timeframe factors** (hospital; calendar year);
- **Maternal health factors** (type of CS; maternal age; hypertension/diabetes; amniocentesis; N. of obstetric checks;

N. of US scans performed; labour induction; labour analgesia; neonatal status; presentation; pre-delivery LoS);

- **Child’s fragility factors** (Apgar score at 5 minutes; ICU admission; multiple birth);
- **Child’s size factors** (gestational age; birthweight; placenta weight);
- **Obstetric history factors** (parity; history of CS)
- **Socio-demographic factors** (paternal age; mother nationality; mother’s educational level)
